# Smooth muscle Cxcl12 contributions to vascular remodeling in flow and hypoxia-induced pulmonary hypertension

**DOI:** 10.1016/j.jbc.2025.110207

**Published:** 2025-05-08

**Authors:** Timothy Klouda, Savas T. Tsikis, Thomas I. Hirsch, Yunhye Kim, Yan Li, Julia Gaal, Zhiyue Zhao, Ingeborg Friehs, John Y.-J. Shyy, Benjamin A. Raby, Mark Puder, Ke Yuan

**Affiliations:** 1 Division of Pulmonary Medicine, Boston Children's Hospital, Harvard Medical School, Boston, Massachusetts, USA; 2 Vascular Biology Program, Boston Children's Hospital, Harvard Medical School, Boston, Massachusetts, USA; 3 Department of Surgery, Boston Children's Hospital, Harvard Medical School, Boston, Massachusetts, USA; 4 Department of Cardiac Surgery, Boston Children's Hospital, Harvard Medical School, Boston, Massachusetts, USA; 5 Division of Cardiology, Department of Medicine, University of California, San Diego, La Jolla, California, USA

**Keywords:** pulmonary hypertension, shear stress, smooth muscle cells, left pneumonectomy, CXCL12

## Abstract

Patients with congenital heart disease causing significant left-to-right shunting of blood are at risk of developing pulmonary arterial hypertension (PAH). However, the underlying mechanism by which pulmonary overcirculation and shear stress drive vascular remodeling remains poorly understood. Our study established a “two-hit” murine model of severe pulmonary hypertension by combining left pneumonectomy (LP) and chronic hypoxia (LP/Hx). Using transgenic reporter lines, immunofluorescence staining, and advanced microscopy, we conducted cell-lineage tracing for vascular cells, including smooth muscle cells (SMCs), endothelial cells, and pericytes. Our findings reveal that SMCs are key contributors to the distal arteriolar remodeling following LP and LP/Hx. PCR analysis of SMCs isolated from LP/Hx animals demonstrated upregulation of markers associated with contractility, proliferation, and *Cxcl12* expression. Consistently, CXCL12 was found to be overexpressed in the SMC layer of pulmonary vessels from patients with PAH–congenital heart disease. Lastly, *in vitro* experiments with healthy human pulmonary artery SMCs showed that laminar shear stress induces CXCL12 upregulation. These findings provide novel insights into the role of SMCs in flow-induced vascular remodeling and their mechanosensitive response to shear stress. This murine model of severe pulmonary hypertension is a valuable tool for future research and developing targeted therapeutics for patients with PAH.

Pulmonary arterial hypertension (PAH) is a devastating, chronic, and progressive disease characterized by remodeling of the distal arterioles and elevated pulmonary artery pressures (1, 2). A significant histopathological feature of PAH is neointimal hyperplasia of the distal arterioles, driven by dysregulated proliferation of smooth muscle cells (SMCs) and apoptosis-resistant endothelial cells (ECs) (3). Pulmonary overcirculation is implicated in vascular remodeling and can lead to the development of pulmonary hypertension (PH). Notably, 37.9% of the patients develop a mild/moderate increase in pulmonary artery pressures after major lung resection, and 3.4% develop severe PH (4, 5, 6). Increased pulmonary blood flow and shear stress often result from intra- or extra-cardiac shunting, especially in patients with specific types of congenital heart disease (CHD) leading to pulmonary overcirculation. Approximately 3 to 10% of the patients with CHD, particularly those with large left-to-right shunts such as a ventricular septal defect (VSD) or atrioventricular canal defect, are at risk of developing PAH (7, 8, 9). Despite advances in the care of patients with CHD, disease-modifying therapies for PAH–CHD are unavailable, and its diagnosis is associated with substantial morbidity, mortality, and increased healthcare costs (7, 8, 9, 10, 11, 12).

The mechanisms by which pulmonary overcirculation and shear stress drive vascular remodeling in PAH–CHD remain poorly understood (9, 13). Early-stage PAH–CHD is reversible, characterized by medial hypertrophy and increased muscularization of the distal arterioles, while late-stage disease can lead to irreversible intimal proliferation and plexiform lesions (14, 15). Studies investigating the pathogenesis of PAH–CHD have primarily focused on the role and contribution of ECs or circulating ECs (16, 17, 18). Endothelial cells sense and respond to hemodynamic shear stress through complex, spatially distributed mechanotransduction mechanisms that regulate vascular remodeling and contribute to vascular pathology (19). Existing rodent models of PH, including “two-hit” approaches combining left pneumonectomy (LP) with the VEGFR antagonist Sugen-5416 (SUGEN) or monocrotaline (MCT) predominantly in rats (MCT pyrrole for mice), have been employed to study flow-induced PH but have limitations (20, 21, 22, 23, 24). Most notably, these rodent models often fail to replicate the physiological conditions experienced by patients, including those with CHD. A murine model that combines increased flow and hypoxia (Hx) may better recapitulate the clinical presentation of PAH–CHD, enabling a comprehensive study of the underlying mechanisms and evaluation of potential disease-modifying therapies.

Chronic Hx is widely used to induce PH in rodents. The murine model of Hx-induced PH is characterized by an increased presence of Smooth muscle actin (Sma)+ cells on the distal arterioles (25, 26, 27, 28). Similarly, the increased accumulation of SMA + SMCs in the distal arterioles is a prominent histological finding in the vascular lesions of patients with PAH (29). Prolonged periods of Hx in patients with CHD can occur due to sleep disorder breathing, cardiopulmonary anomalies, or complications from surgical interventions (30, 31, 32). In response to Hx, pulmonary blood flow is redirected to better-ventilated regions of the lung by contraction of SMCs, resulting in increased pulmonary vascular resistance and tone (known as hypoxic pulmonary vasoconstriction) (33, 34). However, the synergistic effects of Hx and shear stress on vascular remodeling, as well as the molecular pathways contributing to flow-induced PH, remain largely under investigation.

The chemokine CXCL12 has emerged as a key regulator of cellular responses to injury, inflammation, and angiogenesis. Cxcl12 signaling through its receptor, Cxcr4, promotes SMC proliferation and pericyte recruitment to arterioles (35, 36). We previously demonstrated that Cxcl12 overexpression drove Ng2+ pericytes to gain a SMC-like phenotype and participate in vascular remodeling in Hx-induced PH (37, 38). Upregulated Cxcl12 expression has been documented in multiple animal models of PH, including SUGEN/Hx rats, MCT rats, Hx bovine, and genetically modified prolyl hydroxylase domain-containing protein 2 and von Hippel–Lindau loss of function mice (39, 40, 41, 42, 43, 44, 45, 46, 47). Additionally, clinical PAH samples and animal models of PH reveal heightened CXCL12 activity in ECs, pericytes, and adventitial fibroblasts (38, 44, 48). Despite the growing literature and evidence, the role of Cxcl12 in flow-induced vascular remodeling remains unclear.

In this study, we investigated the role of SMCs in an augmented murine model of severe PH combining increased pulmonary blood flow *via* LP and chronic Hx. Our key findings include the following: (1) the development of a murine PH model with a high right ventricle systolic pressure (RVSP), right ventricle hypertrophy (RVH) and increased vascular remodeling of distal arterioles; (2) identification of SMCs as a key contributor to vascular remodeling in the LP/Hx mouse model by using IF staining and a transgenic reporter mouse line (*Acta2-CreERT2::R26-mTmG*); (3) upregulation of Cxcl12 and markers of contractility and proliferation in SMCs isolated from LP/Hx mice; (4) elevated expression of CXCL12 in the distal vasculature of patients with PAH-CHD (VSD); and (5) increased CXCL12 expression in human pulmonary arterial SMCs (hPASMCs) exposed to laminar shear stress. Future integration of this model with genetically modified mice, such as gene knock-out or overexpression and fate mapping, will further elucidate pathophysiological mechanisms and potential treatments.

## Results

### LP combined with hypoxia results in exacerbated PH and RVH

**Figure 1:**
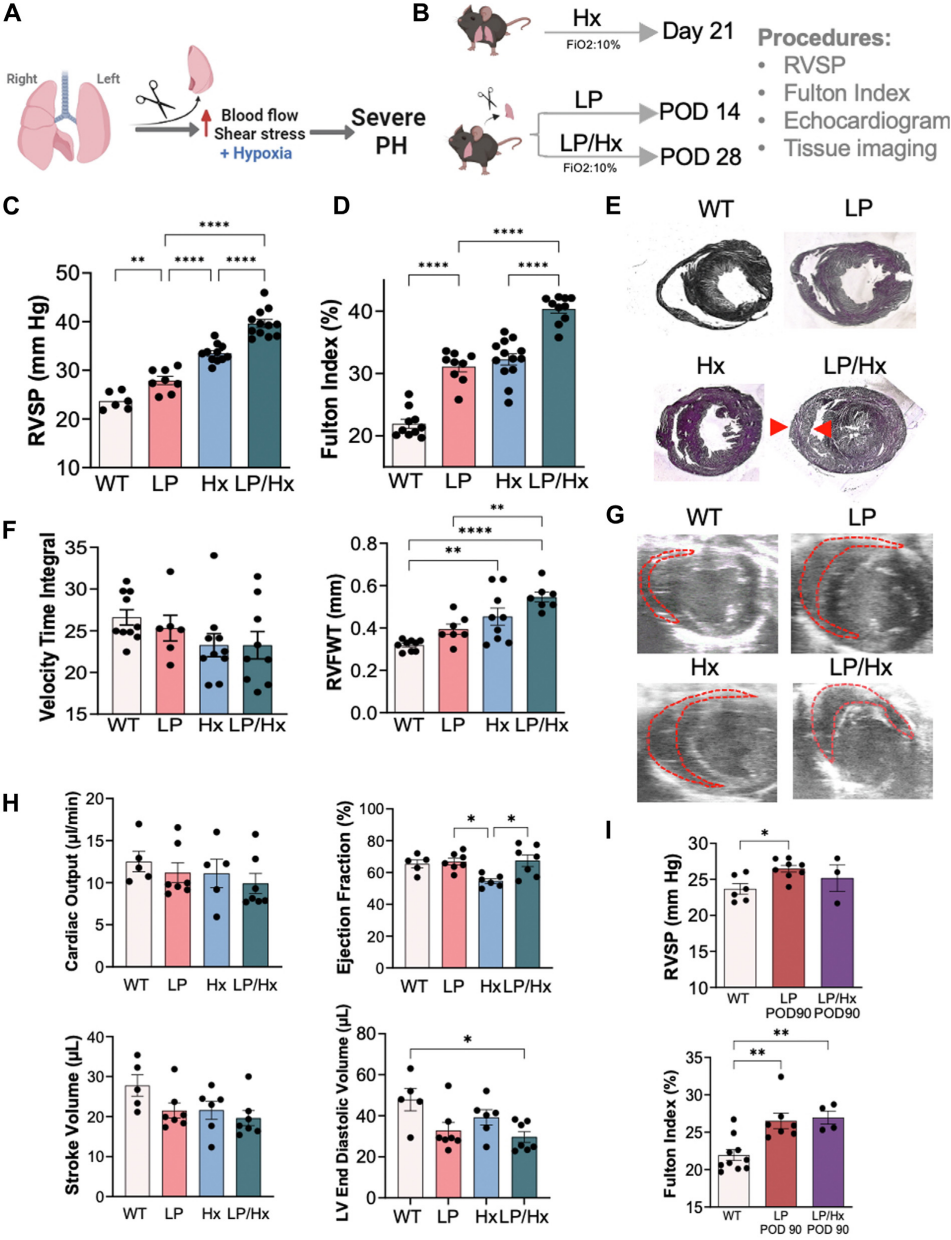
**Left pneumonectomy combined with hypoxia results in exacerbated PH and RVH.***A*, the illustration shows the hypothesis that increased blood flow and Hx result in severe PH. *B*, schematics showing experimental designs. *C*, right ventricular systolic pressure (RVSP) measurements were conducted for control (n = 6), LP (n = 11), Hx (n = 8), and LP/Hx (n = 12) mice. *D*, Fulton Index (FI) was calculated for right ventricle hypertrophy (RVH) in control (n = 10), LP (n = 13), Hx (n = 9), and LP/Hx (n = 10) mice. *E*, cross-sections of hearts were stained with H&E, demonstrating a thickened RV wall (*red* arrowheads) in LP/Hx mice. *F*, right ventricle (RV) echocardiographic measurements were conducted in control (n = 9–10), LP (n = 7–8), Hx (n = 9–10), and LP/Hx (n = 7–10) mice. *G*, representative echocardiogram images of the RV in end-diastole were captured with the RV outlined in *red dashedlines*. *H*, left ventricle echocardiographic measurements were conducted in control (n = 5), LP (n = 7), Hx (n = 5–6), and LP/Hx (n = 7) mice. *I*, RVSP and FI measurements in control (n = 6–10), LP POD 90 (n = 7–8), and LP/Hx recovery mice (n = 3–4). Each *dot* represents an individual mouse. Statistical analysis was performed using one-way ANOVA. Error bars demonstrate mean ± standard error. ∗*p* < 0.05, ∗∗*p* < 0.01, and *∗∗∗∗p* < 0.0001 indicates statistical significance.

We hypothesized that exposure to Hx following pulmonary overcirculation induced by LP would exacerbate PH and remodeling of distal arterioles (Fig. 1*A*). To test this hypothesis, adult mice were exposed to 3 weeks (wks) of Hx 7 days after LP. RVSP measurements were obtained after exposure to Hx on postoperative day (POD) 28 and in experimental mice for comparison (LP, Hx, and control) (Fig. 1*B*). POD 14 was chosen as the endpoint for normoxic LP measurements as our prior experiments have demonstrated no significant difference in PH measurements on POD 14 compared to POD 28, and compensatory lung growth (CLG) is nearly complete by POD 8 (49). Additionally, POD 14 is a commonly selected time point in experiments utilizing the LP mouse and allows a comparison between models (50, 51, 52). LP/Hx mice did not experience any additional postoperative mortality after POD 7, with overall survival rates after LP similar to our prior studies (50). RVSP measurements were significantly elevated in LP/Hx mice compared to Hx (39.6 ± 0.8 versus 33.5 ± 0.6 mm Hg, *p* < 0.0001) and LP alone (27.9 ± 0.8 mm Hg, *p* < 0.0001) (Fig. 1*C*). Similarly, the Fulton index (FI), a measurement of RVH, was markedly increased in LP/Hx mice compared to Hx (40.4 ± 0.7 versus 32.3 ± 0.9%, *p* < 0.0001) and LP (31.1 ± 0.8%, *p* < 0.0001) (Fig. 1*D*). H&E staining of *postmortem* heart sections revealed a thickened right ventricle (RV) wall after LP/Hx compared to LP and Hx alone (Fig. 1*E*).

Echocardiogram was performed to assess both RV and left ventricle (LV) function in experimental animals. The pulmonary artery velocity time integral (VTI), an indicator of pulmonary blood flow and disease severity, was lower in LP/Hx mice than in controls, though the difference was not statistically significant (23.3 ± 1.6 *versus* 26.6 ± 0.9, *p* = 0.30) (53, 54, 55, 56). However, RV free wall thickness was elevated in LP/Hx mice compared to LP (0.55 ± 0.02 *versus* 0.39 ± 0.2 mm, *p* = 0.0054) and control mice (0.32 ± 0.01 mm, *p* < 0.0001) (Fig. 1*F*). Echocardiogram images and videos of the short axis revealed profound RV dilation in LP/Hx mice compared to Hx and LP alone, as illustrated in Figure 1*G* and Videos S1–S4. In contrast, LV measurements in LP/Hx mice showed no significant change in cardiac output (9.9 ± 1.2 *versus* 12.5 ± 1.2 μl/min, *p* = 0.52), ejection fraction (67.5 ± 3.6 *versus* 65.5 ± 2.6%, *p* = 0.96), and stroke volume (19.6 ± 1.9 *versus* 27.8 ± 2.7 μl, *p* = 0.071) compared to controls. However, LV end-diastolic volume was significantly decreased in LP/Hx mice (29.7 ± 2.6 *versus* 47.9 ± 5.4 μl, *p* = 0.020) when compared to controls (Fig. 1*H*). While echocardiographic measurements are inherently variable and subjective, our data collectively demonstrated that LP/Hx mice developed more severe PH with signs of RV dysfunction than Hx alone, whereas LV function remained preserved.

To test the reversibility of this phenotype, we measured the RVSP and FI in LP/Hx mice on POD 90 after returning them to normoxic conditions for 62 days (LP/Hx POD 90). RVSP measurements (25.2 ± 1.8 *versus* 26.5 ± 0.5 mm Hg, *p* = 0.59) and FI calculations (27.0 ± 0.9 *versus* 26.5 ± 1.0, *p* = 0.95) were similar between LP/Hx POD 90 and LP POD 90 mice, suggesting some reversibility of LP/Hx mice when returned to normoxic conditions (Fig. 1*I*) (49).

### LP/Hx results in exacerbated vascular remodeling and accumulation of Sma + cells on distal arterioles

**Figure 2:**
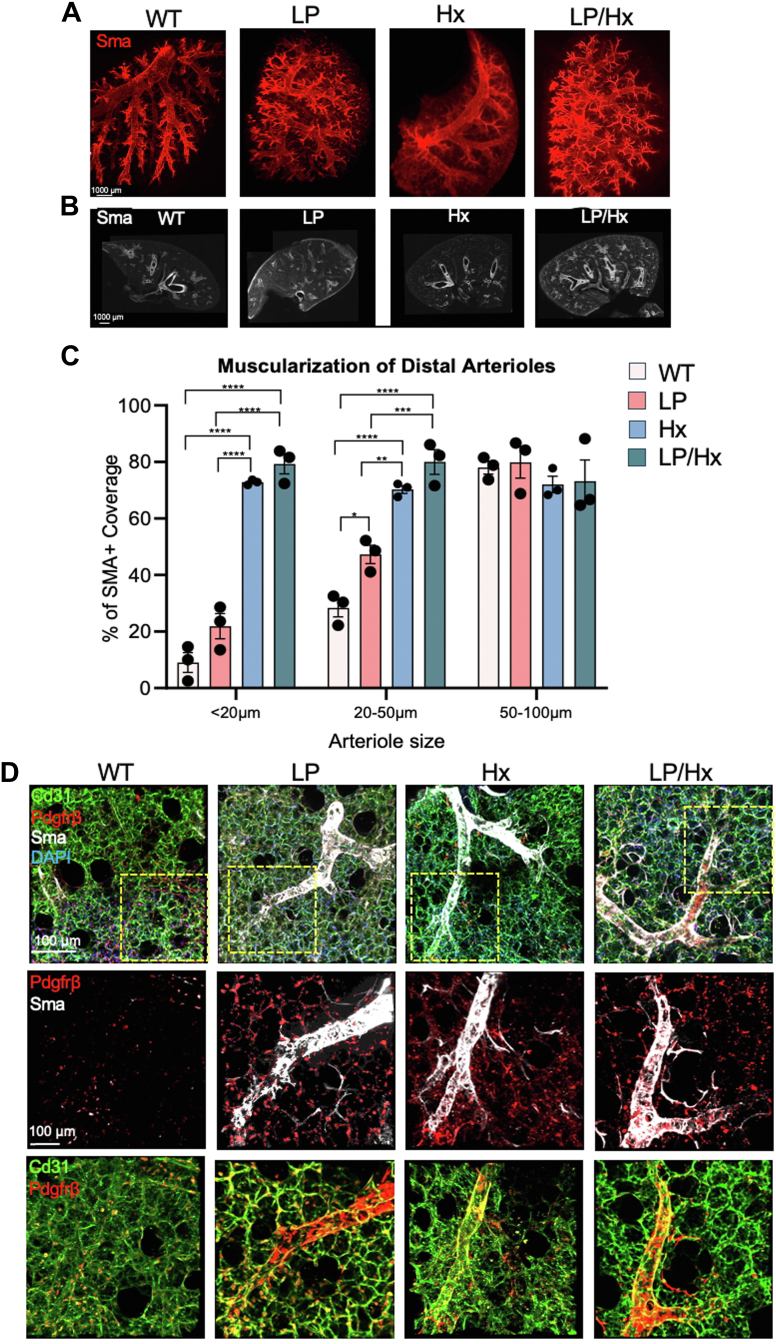
**LP/Hx results in exacerbated vascular remodeling and accumulation of Sma + cells on distal arterioles**. *A*, tissue clearing and deep whole lung lobe staining were performed on experimental mouse lungs. Sma is in *red*. Scale bar represents 1000 μm. *B*, precision cut lung slices (PCLSs) from control, LP, Hx, and LP/Hx mice showing increased accumulation of Sma (*white*) on the arterioles of LP/Hx mice. Scale bar represents 1000 μm. *C*, graph shows the percentage of distal arterioles (small: <20 μm, medium: 20–50 μm, large: >50 μm) with >50% Sma coverage. N = 3 for each experimental group. Each *dot* represents one PCLS inspected from a unique animal. *D*, PCLSs from experimental animals stained for Cd31 (endothelium, *green*), Sma (SMCs, *white*), Pdgfrβ (pericytes, *red*), and DAPI (*blue*). Scale bar represents 100 μm. *Yellow* boxes demonstrate increased magnification. Error bars demonstrate mean ± standard error. Statistical analysis was performed with one-way ANOVA. *∗p* < 0.05, *∗∗p* < 0.01 *∗∗∗p* < 0.001, and *∗∗∗∗p* < 0.0001 indicates statistical significance.

With our hemodynamic measurements revealing LP/Hx mice developed exacerbated PH and RV dysfunction compared to LP and Hx alone, we investigated the extent of vascular remodeling in the distal arterioles using immunofluorescence (IF) staining. Whole lung deep tissue clearing using iDISCO and light-sheet microscopy demonstrated a significant increase in Sma expression within the distal vasculature of LP/Hx mice compared to LP or Hx alone (Fig. 2*A*). To further quantify this remodeling, precision-cut lung slices (PCLSs) were stained for Sma and inspected with confocal microscopy. Minimal Sma + cells were found on the distal arterioles (<50 μm) of controls, whereas all three experimental models (LP, Hx, LP/Hx) revealed an increased accumulation of Sma in the distal arterioles. (Fig. 2*B*). Quantification of the distal vasculature from PCLSs of LP/Hx mice showed a significantly increased proportion of small arterioles (<20 μm) expressing Sma compared to LP (79.3 ± 3.5 *versus* 21.9 ± 4.4%, *p* < 0.0001) but no difference compared with Hx (72.8 ± 0.5%, *p* = 0.55). A similar trend was seen in medium-sized vessels (20–50 μm), with increased Sma expression in LP/Hx tissue compared to LP (80.0 ± 4.3 *versus* 47.3 ±0 .3.3%, *p* < 0.0001) and no significant difference compared to Hx alone (70.0 ± 1.3%, *p* = 0.22). As expected, there was no difference in the amount of Sma in large vessels (>50 μm) across all four experimental groups (Fig. 2*C*). Additional IF staining of PCLSs for ECs (Cd31), pericytes (Pdgfrβ), and SMCs (Sma) was performed to investigate the contribution of vascular and mural cells to flow-induced vascular remodeling. LP/Hx mice revealed increased cells accumulated on distal arterioles co-expressing Pdgfrβ and Sma compared to Hx, LP, and control mice (Fig. 2*D*, top row). Representative images with increased magnification can be outlined in the yellow boxes (Fig. 2*D*, bottom rows).

### Fate mapping using *Acta2-CreERT2::R26-mTmG* mice suggests SMC origin cells contribute to increased muscularization on distal arterioles

**Figure 3:**
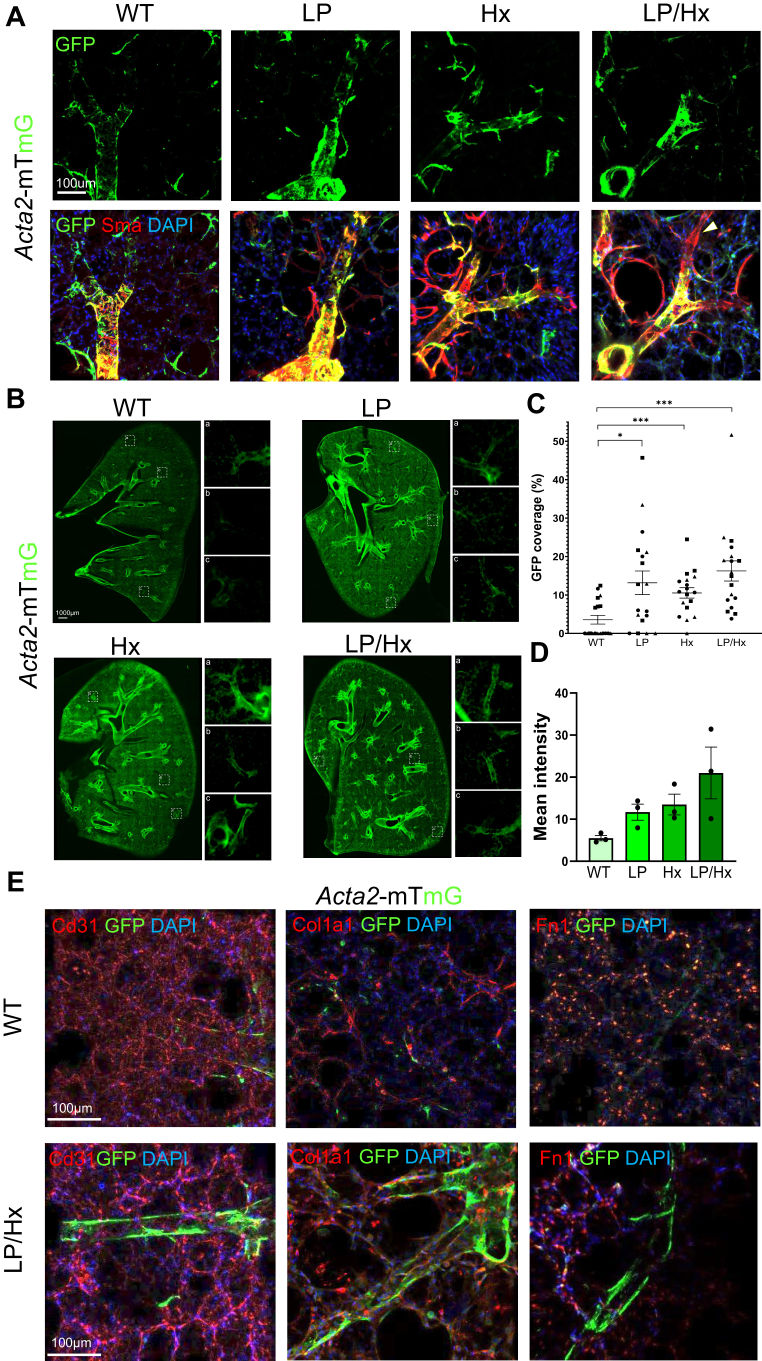
**Fate mapping using *Acta2-CreERT2::R26-mTmG* mice suggests SMC origin cells contribute to increased muscularization on distal arterioles.***A*, fate mapping experiments were conducted for *Acta2-mTmG* mice in control, LP, Hx, and LP/Hx mice. IF staining was performed for Sma (*red*) and DAPI (*blue*). Notice both the colocalization of GFP + cells and Sma staining in LP and LP/Hx mice and the presence of GFP-, Sma + cells increased in the distal vasculature of LP/Hx mice compared to Hx and LP (*white arrow*). Scale bar represents 100 μm. *B*, precision cut lung slices (PCLS) from Acta2-mTmG experimental mice suggest the accumulation of endogenous GFP (*green*) in LP/Hx mice. The *white* boxes indicate the magnified areas in panels to the right (*C, D*), which are used for quantification. Scale bar represents 1000 μm. *C*, quantifications of endogenous GFP coverage in *Acta2-mTmG* control, LP, Hx, and LP/Hx mice. Each *dot* represents one vessel inspected from a PCLS. For each condition, six vessels were inspected from three (n = 3) biological samples. *D*, mean intensity was quantified for GFP in control, LP, Hx, and LP/Hx mice. N = 3 for each group. Each *dot* represents the average of six vessels inspected from a unique mouse. *E*, additional IF staining of *Acta2-mTmG* control and LP/Hx mice for Cd31 (*red*, *left*), Col1a1 (*red*, *middle*), and Fibronectin (*red*, *right*). DAPI (*blue*). Scale bar represents 100 μm. Error bars demonstrate mean ± standard error. Statistical analysis was performed with the Mann-Whitney *t* test. ∗*p* < 0.05, ∗∗∗*p* < 0.001 indicates statistical significance.

Due to the increased accumulation of Sma+, Pdgfrβ+ cells in the distal arterioles of PCLSs from LP/Hx mice, we employed fate-mapping techniques with transgenic mice to further elucidate the roles of SMCs, ECs, and pericytes in vascular remodeling secondary to pulmonary overcirculation. To assess the response of pulmonary SMCs to increased blood flow and Hx, we utilized *Acta2-CreERT2::R26-mTmG* (*Acta2-mTmG*) mice (57). After intraperitoneal (IP) tamoxifen injections, SMCs were appropriately labeled with GFP. PCLSs were stained for Sma (red) to evaluate the labeling efficiency. In *Acta2-mTmG* control animals, GFP+, Sma + cells were predominantly found in arteries >100 μm in size. Following LP, Hx, and LP/Hx, the number of GFP+, Sma + cells increased in small arterioles (50 μm), indicating that SMCs were a source of muscularization and remodeling in flow and Hx-induced PH (Fig. 3*A*). Notably, we observed a subpopulation of GFP-, Sma + cells on distal vasculature in *Acta2-mTmG* LP/Hx mice (Fig. 3*A*, white arrow).

To better quantify the contribution of Sma + SMCs to vascular remodeling, we analyzed PCLSs from *Acta2-mTmG* experimental mice and measured GFP expression in the distal vasculature. Visual inspection of PCLSs for endogenous GFP expression from *Acta2-mTmG* mice with confocal microscopy demonstrated an increase in GFP outlining the distal vasculature in LP/Hx mice compared to controls (Fig. 3*B*). *Acta2-mTmG* LP/Hx mice showed a higher percentage of GFP covering the distal vasculature compared to control mice (16.3 ± 2.6 *versus* 3.6 ± 1.1%, *p* < 0.0001). A similar increase was also seen in LP mice (13.2 ± 3.1; *p* = 0.011); however, no difference was demonstrated between LP/Hx, Hx, and LP mice (Fig. 3*C*). Lastly, the mean intensity of GFP on the distal vasculature in LP/Hx mice was calculated and found to be increased by approximately four-fold compared to control mice (21.0 ± 6.2 *versus* 5.5 ± 0.64, *p* = 0.100) and two-fold increase compared to LP mice (11.7 ± 1.9, *p* = 0.100) (Fig. 3*D*). Of note, inspection of PCLSs from experimental *Acta2-mTmG* mice demonstrated significant variability, as demonstrated in calculations and quantifications performed. To confirm the specificity of GFP labeling in SMCs, additional IF staining was performed for fibroblasts (collagen: Col1a1 and fibronectin: Fn1) and ECs (Cd31), revealing minimal co-expression of any staining with GFP + cells in control mice (Fig. 3*E*).

To determine whether ECs and pericytes directly contribute to flow-induced vascular remodeling, similar experiments were performed in EC fate mapping mice (*Cdh5-CreERT2::R26-mTmG,* or *Cdh5-mTmG*) and pericyte fate mapping mice (*Cspg4-CreER::R26-tdTomato*, or *Cspg4-tdT*) (58, 59, 60). Staining for control antibodies in all three transgenic mice (Sma for SMCs, Cd31 for ECs, and Pdgfrβ for pericytes) can be seen in Fig. S1*A*. Inspection of PCLSs revealed no obvious coexpression of ECs (GFP) with Sma staining after LP nor were there notable differences between LP/Hx and Hx mice. In contrast, pericytes (tdT) demonstrated increased surface area following LP and some tdT + cells translocated to distal vessels upon exposure to Hx coexpressing Sma (Fig. S1*B*). However, the SMC coverage of the distal arterioles in Hx-exposed mice, confirmed by both antibody staining and lineage tracing with GFP, was more dominant than the Sma+ Ng2+ cells seen in *Cspg4-tdT* Hx mice. These findings suggest that SMCs are key contributors to vascular remodeling in flow-induced PH. We next sought to determine the underlying mechanism driving these SMC changes.

### Pulmonary overcirculation combined with hypoxia leads to upregulation of Cxcl12 and an increase in contractility/proliferative cell markers in SMCs

**Figure 4:**
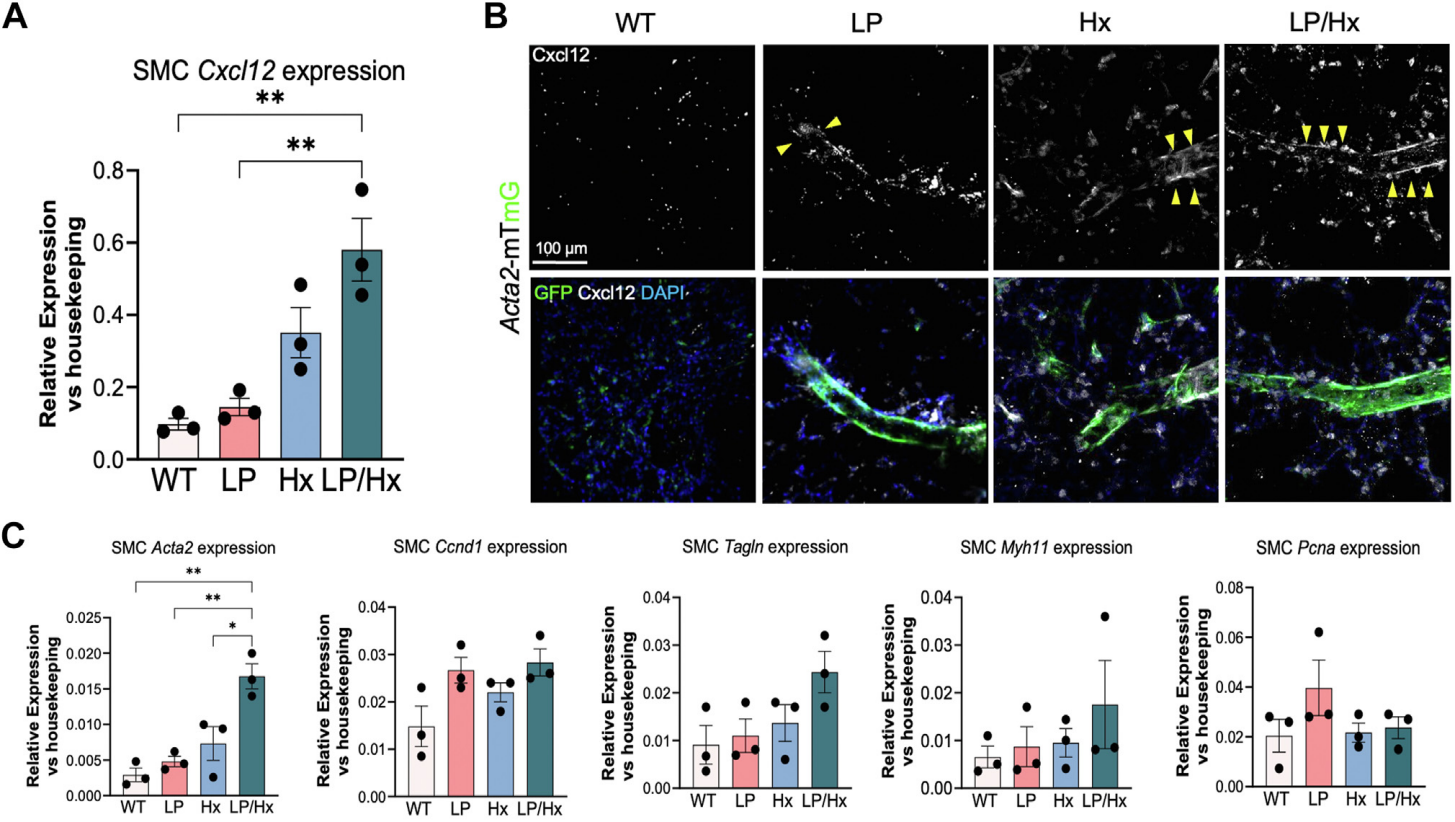
**Pulmonary overcirculation combined with hypoxia leads to upregulation of Cxcl12 and an increase in contractility/proliferative cell markers in SMCs.***A*, real-time qPCR (qRT-PCR) of isolated SMCs (Cd146+) from control, LP, Hx, and LP/Hx mice for *Cxcl12*. The graph shows the relative expression of Cxcl12 compared to the housekeeping gene B2m. N = 3 for each experimental group. *B*, Precision cut lung slices (PCLS) from Acta2-CreERT2::R26-mTmG mice showing increased accumulation of Cxcl12 (*white*) in GFP + cells (SMCs) on distal arterioles (*yellow arrows*) in control, LP, Hx, and LP/Hx mice. Scale bar represents 100 μm. *C*, qRT-PCR of isolated SMCs (Cd146+) from control, LP, Hx, and LP/Hx mice for secretory/contractility (*Acta2*, *Tagln*, *Myh11*) and proliferative (*Ccnd1*, *Pcna*) cell markers. The graph shows the relative expression of each gene over the housekeeping gene B2m. N = 3 for each experimental group. Each *dot* represents a unique sample. Statistical analysis was performed with one-way ANOVA. Error bars demonstrate mean ± standard error. ∗*p* < 0.05, ∗∗*p* < 0.01 indicates statistical significance.

Emerging evidence suggests that high shear stress impairs the proliferation, migration and phenotypic changes of ECs and SMCs (48, 61, 61). Based on fate-mapping results and the known role of Cxcl12 in mural cell-mediated vascular remodeling in response to Hx, we isolated SMCs and ECs from murine lungs using magnetic Dynabeads targeting Cd146 and Cd31. Real-time qPCR (qRT-PCR) from isolated Cd146+ SMCs revealed an upregulation of *Cxcl12* (normalized to housekeeping gene *B2m*) after LP (1.5-fold increase) compared to controls. SMCs isolated from Hx lungs exhibited a mild increase in *Cxcl12* compared to the control (3.6-fold increase, *p* = 0.06) and LP (2.4-fold increase, *p* = 0.13) groups. Interestingly, SMCs from the LP/Hx mouse model, which developed the most severe PH, RVH, and vascular remodeling, displayed the highest *Cxcl12* expression (6.0-fold increase over controls, *p* = 0.0015; 4.0-fold increase over LP, *p* = 0.003; 1.7-fold increase over Hx, *p* = 0.09) (Fig. 4*A*). Similarly, isolated Cd31+ ECs from LP/Hx mice demonstrated a 5.5-fold upregulation of *Cxcl12* compared to controls (*p* = 0.17), with ECs from LP and Hx mice showing a 2.3- and 3.2-fold increase over controls, respectively (Fig. S2*A*). IF staining of PCLSs revealed that Cxcl12 (white) increased around distal arterioles (<50 μm) stained with GFP + SMCs and ECs in all experimental animals (Figs. 4*B* and S2*B*). Additionally, increased Cxcl12 staining was observed diffusely throughout the pulmonary parenchyma, suggesting that cell types other than ECs and SMCs may also contribute to Cxcl12 upregulation in response to increased pulmonary blood flow and Hx.

To further characterize the response of SMCs in the LP/Hx model, qRT-PCR was performed on isolated SMCs to evaluate the expression of contractile (*Acta2, Tagln, Myh11)* and proliferative (*Ccnd1, Pcna*) cell markers. Isolated SMCs from LP/Hx mice demonstrated a significant increase in *Acta2* expression (5.7-fold, *p* = 0.0012) compared to controls, with a modest but not statistically significant increase in the expression of *Tagln* (2.7-fold, *p* = 0.10). Additionally, SMCs from LP/Hx mice displayed elevated expression of *Ccnd1* (1.9-fold, *p* = 0.060) (Fig. 4*C*). Given the established role of angiogenesis-associated genes in vessel growth after LP, we also examined *Vegfa* and *Kdr* expression in ECs (52). However, qRT-PCR on ECs revealed no significant increase in ECs from LP or LP/Hx compared to controls, as *Kdr* was downregulated in LP/Hx ECs (2.0-fold, *p* = 0.040) compared to the control group (Fig. S2*C*).

### Human PASMCs exposed to laminar shear stress result in CXCL12 upregulation

**Figure 5:**
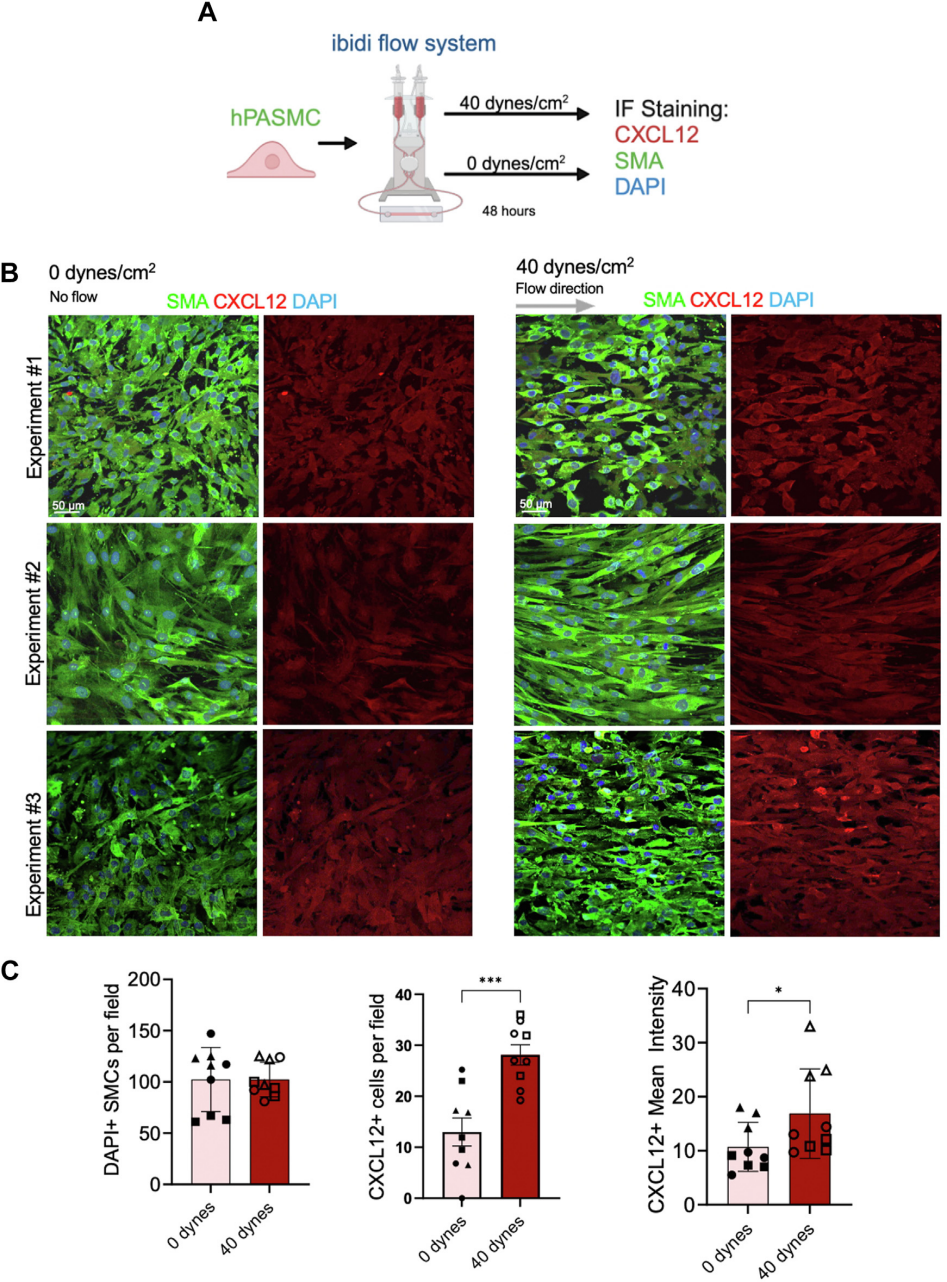
**Healthy human pulmonary artery smooth muscle cells exposed to laminar shear stress result in CXCL12 upregulation.***A*, schematic image shows the proposed flow and cell culture experiments. *B*, IF staining was performed on healthy human pulmonary artery smooth muscle cells (hPASMCs) for SMA (*green*) and CXCL12 (*red*) with and without exposure to laminar shear stress. DAPI (*blue*). Scale bar represents 50 μm. *C*, graphs show immunofluorescence (IF) quantifications of CXCL12 in hPASMCs exposed to laminar shear stress and controls including the percent of overall cells expressing CXCL12 and mean intensity of CXCL12. N = 3 biological samples with three images quantified from each sample. Each symbol represents an image taken from a biological sample. Statistical analysis was performed with the Mann-Whitney test. Error bars demonstrate mean ± standard error. ∗*p* < 0.05, ∗∗∗*p* < 0.001 indicates statistical significance.

After identifying increased *Cxcl12* expression in pulmonary SMCs isolated from LP/Hx mice, we investigated the effects of shear stress on SMCs *in vitro* without exposure to Hx. Healthy hPASMCs were subjected to laminar shear stress (40 dyn/cm^2^) for 48 hours using the ibid flow chamber, and controls were maintained under static conditions (0 dyn/cm^2^) (62) (Fig. 5*A*). IF staining for CXCL12 and SMA was performed to identify cell borders and quantify CXCL12 expression. Confocal microscopy revealed increased expression of CXCL12 (red) in hPASMCs (green) exposed to shear stress (Fig. 5*B*). Quantifications revealed a significant increase in the percentage of CXCL12+, 4′,6-diamidino-2-phenylindole (DAPI) + SMCs exposed to 40 dyn/cm^2^ (28.1 ± 2.0 *versus* 13.0 ± 2.8%, *p* = 0.0008) and an increased mean intensity of CXCL12 (16.9 ± 12.8 *versus* 10.7 ± 1.5, *p* = 0.042) compared to controls (Fig. 5*C*). These *in vitro* results corroborate the molecular data from the LP/Hx mouse model, demonstrating that shear stress of hPASMCs results in increased CXCL12 expression.

### CXCL12 is upregulated in remodeled vessels in PAH–CHD patients

**Figure 6:**
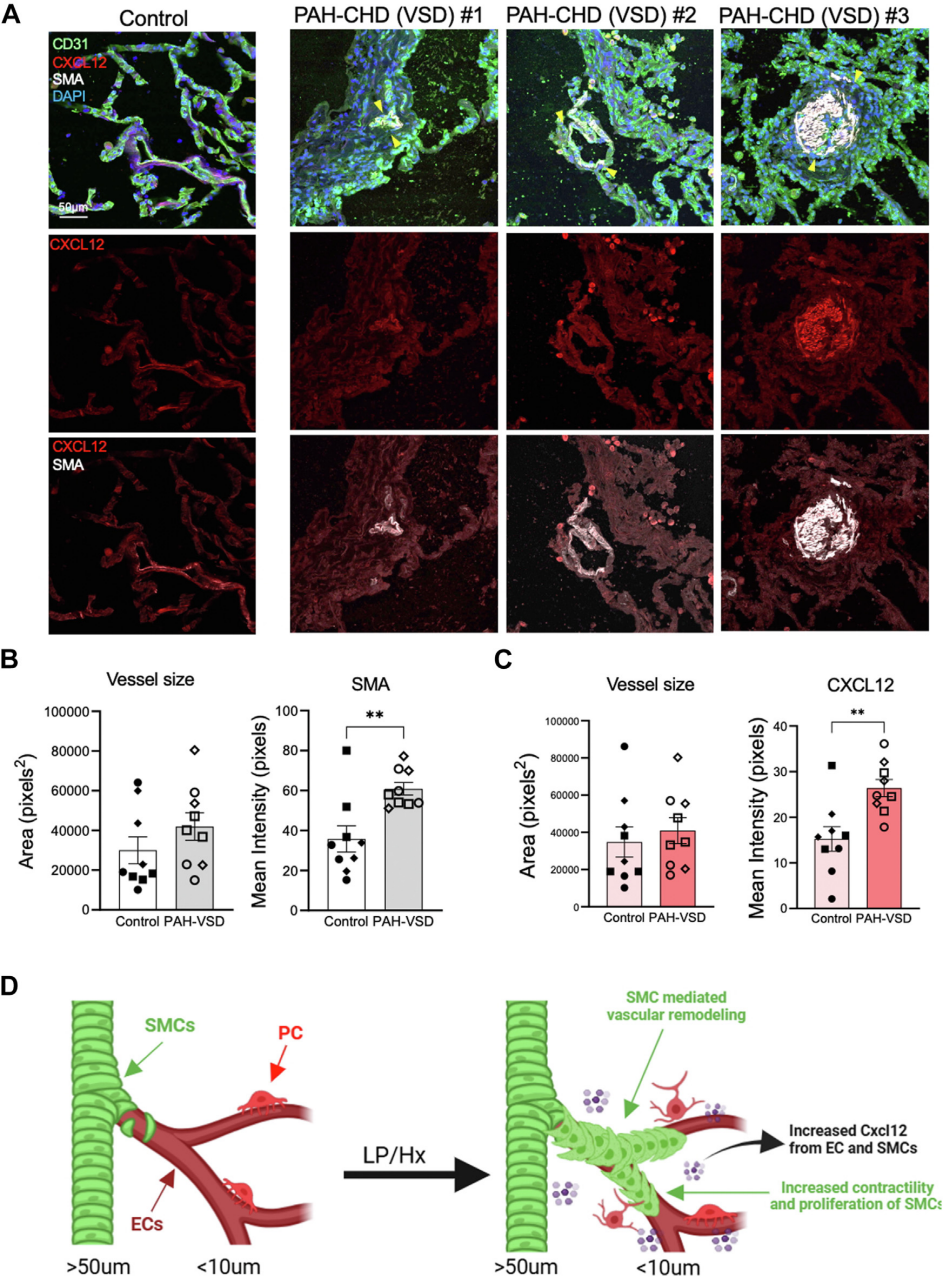
**CXCL12 is upregulated in remodeled vessels in patients with PAH-CHD.***A*, staining of lung tissue from nondiseased patients and patients with PAH-CHD (VSD) for CXCL12 (*red*), SMA (*white*), CD31 (*green*), and DAPI (*blue*). *Yellow* arrows highlight CXCL12 accumulation in remodeled distal vasculature. Scale bar represents 100 μm. *B*, the vessel area and mean intensity of SMA immunofluorescence (IF) staining in human lung samples were quantified for patients with PAH-CHD and nondiseased controls. N = 3 for each group. Each symbol represents an individual calculation of one vessel from a biological sample. For each sample, three vessels were inspected and quantified. *C*, the vessel area and mean intensity of CXCL12 IF staining in lung samples from patients with PAH-CHD and nondiseased controls. N = 3 for each group. Each symbol represents a calculation of one vessel inspected from a biological sample. For each sample, three vessels were inspected and quantified. *D*, schematic illustration shows the role of Cxcl12 and the response of SMCs, ECs, and PCs to increased blood flow and Hx. Statistical analysis was performed with the Mann-Whitney test. Error bars demonstrate mean ± standard error. ∗∗*p* < 0.01 indicates statistical significance.

To assess whether our findings from the LP/Hx murine model correlate with human disease, we examined the expressions of CXCL12 and SMA by performing staining on three explant lung samples from nondiseased patients and three patients with PAH–CHD (PAH secondary to a VSD). Relevant demographics and clinical data, including hemodynamic measurements, are provided in Fig. S3. IF staining of human lung tissues revealed an upregulation of CXCL12 staining around the pulmonary vessels in PAH–CHD patients (Fig. 6*A*). The mean intensity of SMA (60.9 ± 3.1 *versus* 35.8 ± 6.6, *p* = 0.0056) and CXCL12 (26.4 ± 1.9 *versus* 15.3 ± 2.7, *p* = 0.0028) were both increased in the vasculature of patients with PAH–CHD compared to nondiseased controls (Fig. 6, *B* + *C*). Representative images of all six subjects (three nondiseased controls and three with PAH–VSD) along with staining for CXCL12, CD31, and SMA can be seen in Fig. S4. Taken together, we provide evidence that pulmonary overcirculation is associated with SMC CXCL12 upregulation, potentially contributing to vascular remodeling and inflammation observed in human PAH (Fig. 6*D*).

## Discussion

Utilizing a combination of lineage tracing, IF staining, advanced microscopy, and qRT-PCR, we demonstrated that increased blood flow, when combined with Hx, exacerbated PH, RVH, and SMC-driven remodeling of distal arterioles. This process is accompanied by the upregulation of Cxcl12 and increased contractility and proliferative markers in SMCs. This newly developed murine model of severe PH may better represent the pathophysiological conditions underlying vascular remodeling observed in PAH-CHD patients.

Existing animal models of PH, such as the LP/SUGEN rat model, exhibit severe PH and histological features resembling neointimal lesions present in human disease. One study has shown that the combination of LP and MCT pyrrole in mice results in excessive remodeling and endothelial-to-mesenchymal-cell transition (20, 21, 63). In contrast, our group and others found that LP combined with SUGEN or MCT pyrrole in mice did not cause severe PH compared with LP alone (64). Although rat models of PH provide critical insights into disease development, the combination of pulmonary overcirculation and EC antagonists fails to replicate the pathological conditions observed in patients. The exposure of adult mice to chronic Hx induces mild PH and vascular remodeling, characterized by the accumulation of Sma + cells on distal arterioles (25, 26). However, the contribution of SMCs to vascular remodeling secondary to pulmonary overcirculation remains poorly understood, as previous studies have focused on the role of ECs and largely overlooked the role of mural cells (20, 63). Herein, we described the cellular changes induced by pulmonary overcirculation (using LP) and Hx, both individually and combined, using three distinct mouse models (*Acta2-mTmG*, *Cdh5-mTmG,* and *Cspg4-tdT*) that enable lineage tracing. Our results demonstrated that SMCs predominantly contributed to both flow- and Hx-induced vascular remodeling (Fig. 3, *A*–*D*). Interestingly, fate mapping experiments and IF staining revealed that a subset of Sma + cells in the distal arterioles of *Acta2-mTmG* LP/Hx mice were GFP-, suggesting the involvement of a subpopulation of SMCs that was not captured through fate mapping approaches. These cells may directly contribute to the vascular remodeling seen in PH. Additionally, fate mapping cannot determine if the combination of flow and Hx has a synergistic effect on SMC-mediated vascular remodeling. While fate mapping models are invaluable tools for elucidating the roles of cell-specific contribution to PH, IF staining–based quantifications are inherently subjective and require more precise methodologies. To overcome these limitations, more advanced and detailed analyses such as single-cell RNA sequencing and spatial transcriptomics are needed to identify the exact cell populations and molecular pathways driving vascular remodeling observed in the LP and LP/Hx mouse models.

Patients with CHD, including VSDs, are at risk of developing PAH, with the risk being proportional to the size of the defect. For instance, patients with an unrepaired VSD >1.5 cm face a significantly greater risk of Eisenmenger's syndrome (65). Additionally, individuals with CHD may often experience prolonged periods of Hx due to associated cardiopulmonary anomalies, sleep disorder breathing, and postoperative complications (30, 31). The progression to irreversible PH in patients with preexisting pulmonary overcirculation, as observed in specific types of CHD, may result from the combined effects of Hx and increased blood flow. These stimuli drive SMC-mediated vasoconstriction as well as inflammatory and proliferative changes in the pulmonary vasculature. The LP/Hx murine model of PH provides a promising platform for investigating the cellular and molecular mechanisms underlying these processes and the additive effects of pulmonary overcirculation and Hx on vascular remodeling. Furthermore, this model allows the investigation of potential therapeutic interventions aimed at preventing or reversing PH. Although rat models of PH more closely replicate the histopathological features of human PAH lesions, genetic manipulation tools such as the Cre-LoxP system are less commonly available in rats compared to mice. Integrating the LP/Hx mouse model with gene-modulation technologies will further enhance our understanding of disease pathophysiology and advance the development of disease-modifying therapies.

The Cxcl12–Cxcr4 pathway plays a pivotal role in SMC- and pericyte-mediated vascular remodeling in Hx-induced mouse models of PH (36, 38, 66). Inhibition of this pathway through its receptor, Cxcr4, has been shown to reduce SMC proliferation and coverage on distal arterioles (35, 36). However, the role of Cxcl12 in flow-induced vascular remodeling remains unclear. Through IF staining and qRT-PCR, we demonstrated for the first time that isolated SMCs and ECs upregulated *Cxcl12* in both flow- and flow/Hx-induced PH (Figs. 4 and S2). Using SMCs isolated from three different PH models (LP, Hx, LP/Hx), we showed that *Cxcl12* expression in SMCs increased in relation to the severity of PH (Fig. 4*A*). Despite these findings, the direct correlation between pulmonary overcirculation and Hx to vascular remodeling and Cxcl12 upregulation remains uncertain. To address this, we supplemented our animal studies with *in vitro* experiments by exposing hPASMCs to shear stress. Under 40 dyn/cm^2^ of laminar shear stress, healthy hPASMCs exhibited upregulated CXCL12 expression (Fig. 5). Additionally, IF staining of lung tissue from patients with flow-induced PAH (VSD) revealed upregulation of CXCL12 in the SMC layer of the distal vasculature (Fig. 6). These finding suggests that shear stress, in the context of pulmonary overcirculation, drives SMC-mediated Cxcl12 expression, contributing to vascular remodeling and the development of PH. However, the underlying molecular mechanism, whether secondary to EC dysregulation, inflammation, or vasoconstriction, remains unclear and warrants further investigation. To stimulate a more severe scenario, exposing hPASMCs to a higher degree of laminar shear stress, such as 100 dyn/cm^2^, could provide additional insight. Furthermore, our *in vitro* studies did not examine the additive effects of Hx and shear stress. Future *in vitro* experiments subjecting hPASMCs to laminar shear stress and Hx could elucidate potential synergistic effects, further enhancing our understanding of PAH–CHD pathogenesis.

In addition to studying flow-induced PH, the LP mouse model is a well-established model for investigating alveologenesis and CLG. Although the precise mechanisms and cellular response driving CLG remain unclear, previous studies have shown that Cxcl12 is upregulated in the lung parenchyma and plays a role in alveolar regeneration as early as 3 days after LP (67). IF staining in flow- and Hx-induced murine models of PH revealed diffuse upregulation of Cxcl12 in the lung parenchyma (Figs. 4*B* and S2), suggesting other cell types may respond to both stimuli through the Cxcl12–Cxcr4 pathway. However, whether Cxcl12 contributes to abnormal vascular remodeling of the distal arterioles, primes ECs for CLG, or plays a dual role remains uncertain. Our data showed increased *Cxcl12* expression in ECs on POD 14, when the majority of CLG is proposed to be complete (Fig. S2*A*) (68, 69). While early Cxcl12 expression likely supports alveologenesis and angiogenesis, its sustained expression at POD 14 suggests a potential inflammatory role in promoting vascular remodeling. Although angiogenic gene markers were not upregulated in ECs from LP or LP/Hx mice, ECs may still contribute to angiogenesis or vascular remodeling by engaging in cellular crosstalk in response to increased blood flow. To gain deeper insights, future studies leveraging the LP/Hx mouse model should extend beyond fate mapping and qRT-PCR to investigate ECs, their subtypes, and other cell populations involved in vascular remodeling. These studies may shed light on the response of ECs to shear stress and uncover molecular pathways that could lead to therapeutic interventions.

It is important to emphasize that our study selected specific experimental endpoints commonly used in rodent models of PH to facilitate a direct comparison of hemodynamics across all four models. Future studies evaluating mice at alternative or later time points could provide critical insights into the long-term effects of pulmonary overcirculation on vascular remodeling and the reversibility or persistence of PH in these mouse models. Additionally, our study did not include sham animals as controls. In our experience, sham pneumonectomy mice (under normoxia or Hx) exhibited RVSP measurements comparable to those of animals that did not undergo sham pneumonectomy, suggesting a minimal impact on hemodynamics. However, future studies incorporating control and experimental mice, including sham pneumonectomy, across extended and similar time points, will provide valuable insights into the progression of PH and vascular remodeling.

In conclusion, we established a novel and reproducible murine model of severe PH by subjecting mice to 3 weeks of Hx following LP. Using lineage tracing and qRT-PCR, we demonstrated that SMCs partially mediated vascular remodeling driven by increased pulmonary blood flow, which was associated with Cxcl12 upregulation. Integrating this model with gene-modulated strains and advanced bioinformatics approaches will enable future studies to further elucidate the molecular mechanisms and cellular responses underlying flow-induced PH.

## Experimental procedures

Studies in this paper were approved by the Animal Care Committee and the Institutional Guidelines of Boston Children's Hospital. All data is contained within the manuscript and raw images or additional information can be shared upon request from the corresponding author.

### Animals

Experiments adhered to the National Institutes of Health guidelines use of laboratory animals. *Acta2-CreERT2::R26-mTmG* mice (C57BL/6J) were used for SMC fate mapping experiments, *Cdh5-CreERT2::R26-mTmG* mice for EC fate mapping, and *Cspg4-CreER-tdTomato* for pericyte fate mapping. Ear clip–based genotyping was used to identify experimental mice. Male mice were 5 weeks of age or older at the time of tamoxifen injection, surgery, or exposure to Hx. Hemodynamic data of LP at POD 14, LP at POD 90, and control mice were utilized from our group's previous publications (49, 64).

### Tamoxifen administration

*Acta2-CreERT2::R26-mTmG* and *Cdh5-CreERGT2::R26-mTmG* mice were administered 10 mg of tamoxifen *via* IP injection (20 mg/ml dissolved in corn oil) over 5 days. *Cspg4-CreER-tdTomato* mice were injected with 2 mg of tamoxifen once. All mice were at least 5 weeks of age before injection. Transgenic mice were allowed to rest for a minimum of 7 days before undergoing pneumonectomy or being exposed to Hx.

### Hypoxia studies

For chronic Hx experiments, mice were exposed to 10% FiO_2_ *via* Hx chamber with *ad lib* access to food and water for 3 weeks. The chamber was maintained with a continuous mixture of room air and nitrogen gas. The chamber environment was monitored using an oxygen analyzer (Servomex) and CO_2_ was removed with lime granules. The internal chamber was monitored daily for O_2_ concentration, CO_2_ concentration, humidity, and animal welfare. To establish the LP/Hx mouse model, mice were exposed to 3 weeks of Hx 7-9 days after LP. RVSP measurements and tissue harvest were performed after exposure to 3 weeks of Hx. LP/Hx recovery mice were placed back in normoxic conditions after 3 weeks of Hx (POD 28) and hemodynamic measurements were performed 62 days later on POD 90.

### Left pneumonectomy

Animals were anesthetized *via* IP injection with a mixture of ketamine (80–100 mg/kg) and xylazine (5–10 mg/kg). After reaching an appropriate level of sedation, mice were placed on a vertical platform and a light-source was used to transilluminate the vocal cords. Intubation was achieved with a 22-gauge angiocatheter (Becton Dickinson) and animals were ventilated at 180 breaths/min using a rodent ventilator (MiniVent Ventilator; Harvard Apparatus). After adequate hair removal to the animals’ left thorax and appropriate asepsis technique, a left anterior mid-axillary incision was made extending from the axilla to the costal margin (70, 71). The thoracic cavity was entered at the fifth intercostal space and the left lung was carefully lifted through the incision and ligated using a 4-0 silk suture (Ethicon). The rib cage and thoracic cavity were then closed with a 4-0 PDS suture (Ethicon). The skin was closed using a 4-0 nylon suture (Ethicon). Mice were provided with appropriate analgesia and monitored daily for signs of pain and respiratory distress during the postoperative period.

### Mouse lung tissue clearing and preparation of precision cut lung slices

After euthanasia, mice were placed in the supine position, and the abdominal and thoracic cavity was exposed through a midline incision. The sternum was then dissected to expose the contents of the mediastinum. The abdominal aorta was then located and cut. Next, a butterfly needle was inserted into the RV, and the heart and lung slowly perfused with 15 cc of 1× PBS to flush the red blood cells from the circulatory system. Once the lung lobes were white in appearance, the trachea was located and cannulated. Lungs were inflated with 2% low-melting point agarose. After adequate inflation, the trachea was tied, and the mediastinal contents were removed and placed in ice-cold 1× PBS to solidify the agarose. The lung tissue was then fixed in 4% paraformaldehyde (PFA) at 4 °C overnight, followed by washing in 1× PBS for an additional day. Lung lobes were then separated and sectioned (Leica VT1000 S) at a thickness of ∼300 μm for future IF staining experiments and confocal microscopy.

### Morphometric analysis of vascular remodeling and GFP expression in *Acta2-mTmG* mice

PCLS from experimental mice (control, LP, Hx, LP/Hx) underwent IF staining for endothelium (Cc31) and Sma. One stained tissue from each mouse was scanned with a confocal microscope (880 laser scanning confocal with Fast Airyscan) using the Z-stack application to visualize the 3D architecture of the pulmonary vasculature (Cd31+ staining) across different diameters (<20 μm, 20–50 μm, and >50 μm). Each identified vessel was analyzed for Sma coverage, defined as greater than 50% of the vessel costained with Sma. PCLSs were scanned entirely from top to bottom to ensure comprehensive analysis. The percentage of arterioles expressing Sma was quantified for each group (N = 3 per group).

Spiral images of PCLS from *Acta2-mTmG* mice (control, LP, Hx, and LP/Hx) were captured using a Leica Thunder Imager under 20× magnification. To quantify the location and GFP coverage within the distal vasculature, six randomly selected vessels with a diameter between 20 and 40 μm from the lung parenchyma were identified with Cd31 staining. Laser intensity for each sample was standardized across samples using the large airways as a reference point for control. A region of interest (30 μm × 100 μm) for each image was selected based on the strongest fluorescent and GFP expression was measured, and the area (width vs length) of coverage was quantified. GFP+ coverage (Sma + cells) on each vessel was calculated by dividing the estimated GFP+ area by the total region of interest area (3000 μm^2^) and expressed as a percentage.

To calculate the mean intensity of GFP for each sample, the same six images used for percent coverage calculation were analyzed. A portion of each vessel was manually traced in Image J based on GFP expression to define the vessel borders. The mean gray value of the traced area was then calculated. The average intensity across the six vessels was determined for each sample.

### Immunofluorescence staining

PCLSs were blocked with 5% goat or donkey serum in 0.5% Triton X-100/PBS (PBS-T) for 1 hour. Samples were then incubated in the dark with primary antibodies for 1 to 2 days at 4 °C. After incubation, tissue was washed three times in 1× PBS followed by incubation with secondary antibodies overnight in the dark at 4 °C. PCLSs were again washed and placed on microscope slides with mounting media containing DAPI (Vector Labs). All images were captured using a Zeiss confocal 880 Airyscan 2 and processed by Aivia software.

The following antibodies were used for IF staining:

Mouse-anti-mouse/human SMA-647 (1:50; Santa Cruz, sc-32251).

Rat-anti-mouse CD31 (1:100; BD-Pharmingen, 553,370).

Rabbit-anti-mouse PDGFRB (1:200; Invitrogen, MA5-15143).

Rabbit-anti-mouse/human SDF-1 (1:100; Abcam, ab9797).

Rabbit-anti-mouse/human COL1A1 (1:200; Cell Signaling Technology, 39952).

Rabbit-anti-mouse Fibronectin-647 (1:100; Cell Signaling Technology, 26836).

Sheep-anti-human PECAM-1 (1:100. R&D Systems, BAF806).

### Quantification of CXCL12 and SMA expression in human lung samples

After IF staining, human lung samples were imaged using the same magnification and power with a confocal microscope (880 laser scanning confocal with Fast Airyscan). Three vessels were used for quantification from each biological sample and selected using SMA staining as a reference. Similar post-imaging processing was performed on each sample using the best-fit adjustment available with Aivia Software. Images were converted to a JPEG file, and Image J software was used to quantify the area of each vessel chosen for quantification and the mean gray value of CXCL12 and SMA.

### Heart OCT compound

Hearts were removed from the mediastinum after sacrifice and washed thoroughly in 1× PBS. Heart samples were then transferred to 4% PFA overnight at 4 °C. The next morning, samples were washed in 1× PBS for 24 hours and placed in 30% sucrose for a minimum of 24 hours. After sitting in sucrose, samples were embedded in OCT gel and flash-frozen using dry ice. The frozen samples were stored at −80 °C until they were sectioned with a cryostat machine into thin slices. Samples were sectioned until the RV and LV openings became visible and then mounted on microscopy slides for H&E staining. Images were captured with a light microscope (Olympus BX-41 upright microscope, Olympus) at 4× objective.

### Whole mount lung lobe clearing and immunofluorescence staining

After removing the lung from appropriately euthanized animals, the lung lobes were fixed with 4% PFA, washed in PBS containing 0.2% Triton X-100 (PTx.2), and then incubated in 1× PBS/0.2% TritonX-100/20% DMSO overnight. Samples were submerged in 1xPBS/0.1%Tween-20/0.1%Triton X-100/0.1%Deoxycholate/0.1%NP40/20% DMSO overnight. The next day, samples were washed with PTx.2 and placed in permeabilization solution (PTx.2/2.3% glycine/20% DMSO) for 48 hours. Samples were incubated in blocking solution (PTx.2/10% DMSO/5% normal goat serum) for 2 days and then submerged in primary antibodies diluted in PTx.2/0.001% heparin (PTwH)/5% goat serum/10% DMSO for a minimum of 3 days. After incubation, samples were washed in PTwH and placed in secondary antibodies diluted in PTwH/5% goat serum for 3 days. After secondary staining, lung lobes were washed in PTwH and progressively dehydrated in 100% methanol over 2 days and then placed in 33% methanol/66% dichloromethane for 3 hours. Finally, lung samples were washed in dichloromethane and then cleared in dibenzylether. Images were obtained using a light sheet microscope.

### RVSP and FI measurements

To calculate RVSP measurements, mice were anesthetized using isoflurane (2.0% in 2LPM O_2_) administered through a nose cone. After reaching an appropriate level of anesthesia, a small incision was made in the skin to the right of the trachea. The surrounding fat was removed with blunt dissection to isolate and visualize the right jugular vein. The vein was then cannulated using a 1.4 F catheter (Millar Instruments) and carefully and slowly advanced into the RV. The RVSP tracings were analyzed using PowerLab software. RVSP measurements for each animal were obtained by calculating measurements at three different time points separated by a minimum of 30 seconds.

Once RVSP measurements were obtained, mice were euthanized with cervical dislocation. The entire heart was removed from the chest cavity, and the atrium and pericardial fat were dissected away. The interventricular septum between the RVs and LVs was then identified, and the two ventricles were carefully separated. The weight of the RV and LV with the interventricular septum was obtained. The FI was calculated using the following equation: (Right ventricle mass)/(Left ventricle mass + Septum mass).

### Echocardiography

Images of the RV in experimental animals were obtained using transthoracic echocardiogram with VisualSonics Vevo 3100 Software (VisualSonics Inc) and a small rodent transducer (MS-550D, 22–55 MHz). To obtain images, mice were first anesthetized in an induction chamber filled with 2 to 3.0% isoflurane and 100% oxygen. Appropriately anesthetized mice were then placed in the supine position, and a nosecone used to deliver 1.0 to 3.0% isoflurane mixed with oxygen (1.0 L/min) on a stage while echocardiogram was performed. The chest fur of animals was completely removed with a chemical hair remover. For investigation of the RV, representative echocardiogram images of RV dilation were obtained in the short-axis view in end-diastole. Pulse-wave Doppler was used to record pulmonary artery blood flow and obtain velocity time integral measurement. RV free wall thickness was measured in the using M-mode. For LV analysis, VisualSonics LV analysis tool was used on murine heart images in the SAX view. Cardiac output was calculated by multiplying the stroke volume by the manually calculated heart rate. LV measurements include cardiac output, ejection fraction, stroke volume, and LV end-diastolic volume.

### Human cell culture

hPASMCs were cultured on 1% gelatin-coated petri dishes in a humidified incubator maintained at 37 °C with 5% CO2. The cells were grown in growth media (SMC growth media (SMGM-2)) and the culture media was replaced every other day until the cells reached 80 to 90% confluence. Cells were then trypsinized using 0.05% Trypsin-EDTA, followed by the addition of trypsin neutralizer solution to halt the trypsinization process. Trypan blue was applied to assess the total number of viable cells in the suspension before exposure to flow.

### Ibidi slide culture and flow exposure

Human cells were diluted in fresh media to a concentration of 1,600,000 cells/ml. A 30 μl volume (containing 48,000 cells) of the cell suspension was pipetted into the reservoir of an ibidi slide (Cat. No. 80606-90, μ-Slide VI 0.4, ibiTreat - Tissue Culture Treated Coated Polymer Coverslip, ibidi). The ibidi slides were then placed in an incubator at 37 °C with 5% CO_2_. After 2 hours, the cells adhered to the channel, and 120 μl of additional medium was added to the reservoirs. Twenty four hours after filling the reservoirs, cells were exposed to either no-flow conditions (0 dyn/cm^2^) or shear stress (40 dyn/cm^2^) for 48 hours. In the no-flow condition, ibidi slides were placed in an incubator with the medium replaced every 24 hours. For the flow condition, cells were subjected to 40 dyn/cm^2^ shear stress using a commercially available flow pump system for cell culture under flow (ibidi). Both conditions were maintained at 37 °C and 5% CO2 throughout the experiment. After 48 hours, cells were fixed by incubating with 4% PFA at room temperature for 10 min, followed by two washes with PBS.

### Human cell culture immunofluorescence staining

Fixation of the cells with 4% paraformaldehyde was followed by the blocking of nonspecific binding using a buffer (containing 5% normal goat serum and 0.3% Triton X-100 in 1× PBS) at room temperature for 1 hour. After removing the blocking buffer, primary antibodies were applied and incubated overnight at 4 °C. Cells were then washed twice with PBS and incubated with secondary antibodies in dilution buffer at room temperature in the dark for 1 hour. After two washes with PBS, the cells were incubated with a diluted DAPI solution at room temperature for 5 minutes, followed by a single wash with 1× PBS and 1× distilled water. Finally, one drop of ibidi mounting medium (Cat. No: 50001, ibidi) was applied to the reservoir, and the stained slides were stored at 4 °C in the dark until imaging.

The following antibodies were used for staining hPASMCs:

Rabbit-anti-mouse/human SDF-1 (1:100; Abcam, ab9797).

Mouse-anti-human SMA-647 (1:100; Sigma-Aldrich, A2547).

### Quantification of CXCL12 in hPASMCs after flow experiments

After IF staining, samples were imaged using similar magnification and power with a confocal microscope (880 laser scanning confocal). Three images were taken from each biological sample. Similar post-imaging processing was performed on each sample. Images were converted to a JPEG file, and Image J software was used for calculations. First, the total number of DAPI + cells in each image and the number of cells expressing CXCL12 were counted to determine the percentage of cells expressing CXCL12. To measure the intensity of CXCL12, nine cells from each image were selected and outlined using CXCL12 and/or SMA staining for reference. Cells were chosen that subjectively demonstrated the brightest fluorescence of CXCL12. If CXCL12 was not visible in nine cells, random cells were selected. Once outlined, the mean gray value of CXCL12 was calculated using Image J. The nine values obtained in one biological sample were averaged together and presented as one data point in figures.

### Murine cell isolation

Lung tissue was harvested from three experimental mice of the similar condition, combined, and digested using commercial Miltenyi gentle MACS dissociator and lung tissue dissociation kit (Miltenyi Biotec). After digestion, 10 ml of 0.1% FBS/PBS was added to the cell suspension and the solution was passed through a BD Falcon 70 μm strainer (BD Biosciences) to remove any undigested tissue. The solution was centrifuged at 400G for 5 minutes and the cell pellet then resuspended in 1 ml 1× PBS containing Cd45 IgG–coated magnetic Dynabeads and the cell and beads gently rotated at 4 °C for 30 minutes to deplete immune cells. Cd45+ dynabeads were then removed by placing the sample on a magnetic stand and the remaining cells (Cd45-) were incubated in Cd140b-APC antibody (Miltenyi Biotec) for 30 minutes at 4 °C on a shaker. Cells were washed with 1× PBS and then stained with anti-APC beads (Miltenyi Biotec) and allowed to incubate for another 30 minutes at 4 °C on a shaker. The Miltenyi Biotech Automacs Cell Sorter was then used to isolate Cd140b+ cells, which were then digested in 500 μl of Trizol for RNA isolation. Cd45, Cd140b cells were then resuspended in 1 ml of 1× PBS containing Cd146 IgG-coated Dynabeads and gently rotated for 30 minutes at 4 °C. CD146+ beads were then selected with magnetic dissociation, washed with 1× PBS, and digested in 500uL Trizol. The remaining cell solution (Cd45-, Cd140b-, Cd146-) were resuspended in Cd31-IgG–coated Dynabeads and again rotated for 30 minutes at 4 °C. The Cd31 beads were then washed with 1× PBS, selected with magnetic dissociation, and digested in 500 μl Trizol for RNA isolation.

### Real-time quantitative PCR

mRNA expression was determined by quantitative SYBR green real-time PCR. Cellular RNA was extracted using an RNeasy Kit (Qiagen) and converted to cDNA using the High-Capacity cDNA Reverse Transcription Kit with RNase Inhibitor (Thermo Fisher Scientific) following the manufacturer’s protocol. All real-time quantitative PCR studies were run in triplicate. ΔCT determined the difference in mRNA expression against the housekeeping gene *B2m*. Primers were purchased from Integrated DNA Technologies. Biological triplicates were used for data analysis. For each primer, technical triplicates were prepared and the two samples with the closest raw value were selected and averaged together to calculate the final gene Ct value (duplicates). Results can be seen as the relative gene expression compared to the housekeeping gene B2m.

### Statistical analysis

The number of animals per experiment can be found in the figure legends. The number of animals and images used for IF quantifications can be seen in the figure legends and in the corresponding methods section. All data is expressed as mean ± SEM unless otherwise indicated. Statistical significance was determined using either a one-way ANOVA or a Mann-Whitney test. ∗*p* < 0.05, ∗∗*p* < 0.01, ∗∗∗*p* < 0.001, ∗∗∗∗*p* < 0.0001.

## Data availability

All data supporting the findings of this study are available from the corresponding author upon request.

## Supporting information

This article contains supporting information.

## Conflict of interest

The authors declare that they have no conflicts of interest with the contents of this article.

## Supporting information

Supplemental Images

Supplemental Videos

Supplemental Figure 1and video legend
