# Supplementary material for: Smooth muscle Cxcl12 contributions to vascular remodeling in flow and hypoxia-induced pulmonary hypertension
Source: J Biol Chem. 2025 May 8;301(6):110207. doi: 10.1016/j.jbc.2025.110207 (PMC12178926; doi:10.1016/j.jbc.2025.110207)
Supplement: Supplemental Images [file mmc1.pdf]

Supplemental Figure 1

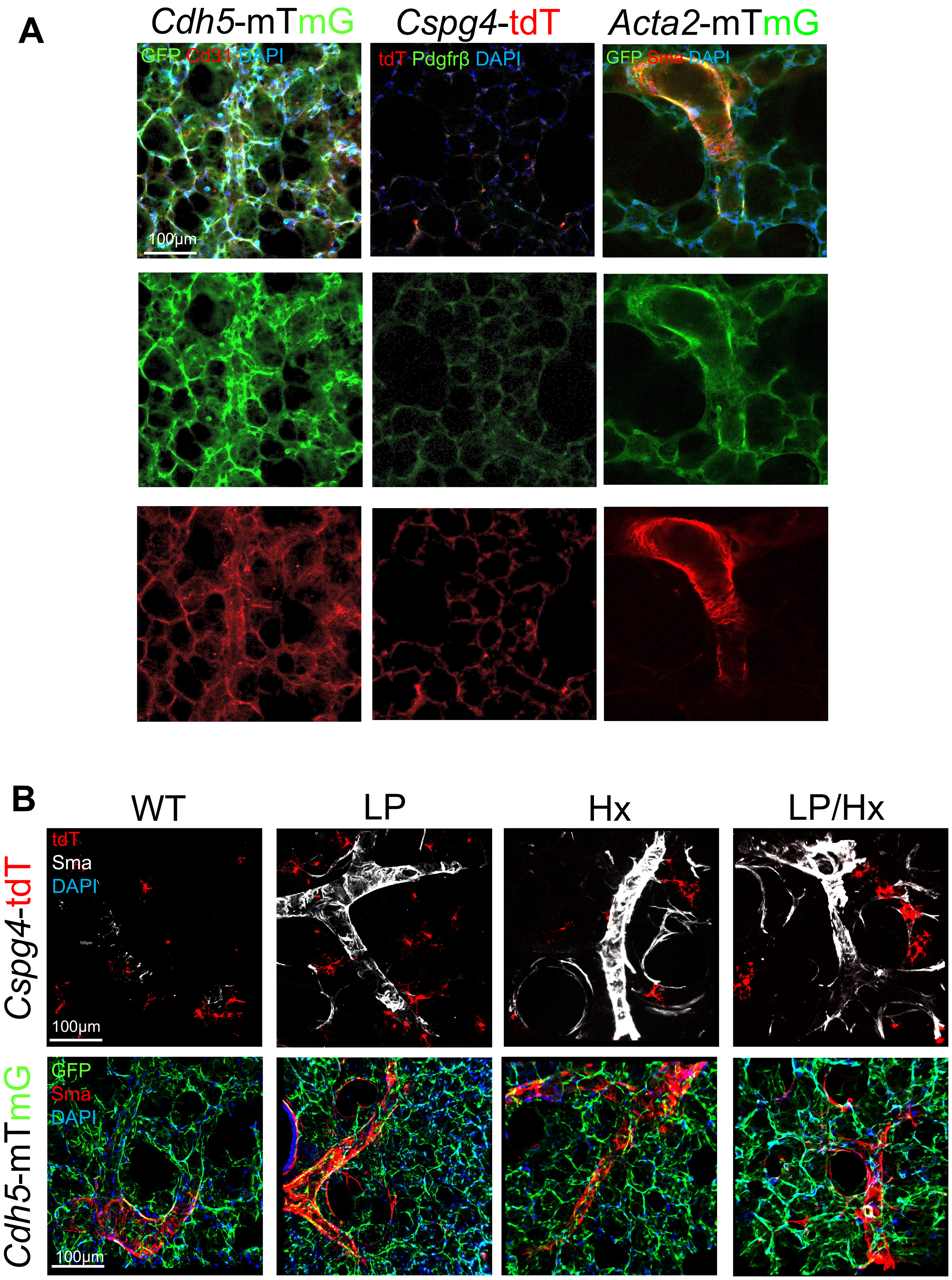

Supplemental Figure 1: EC and pericyte fate mapping experiments with *Cdh5-CreERT2::R26-mTmG and Cspg4-CreER™::R26-tdTomato.*
A, fate mapping experiments in mice with respective control antibodies (*Cdh5-mTmG:* Cd31 (left), *Cspg4-tdT:* Pdgfrβ (middle), and *Acta2-mTmG:* Sma (right)) with endogenous staining (GFP: green, tdT: red). Scale bar: 100 µm.
B, fate mapping experiments with *Cdh5-mTmG* mice and *Cspg4-tdT* mice in control, LP, Hx, and LP/Hx mice. Immunofluorescent (IF) staining was performed for Sma and DAPI (blue). Scale bar: 100 µm.

Supplemental Figure 2

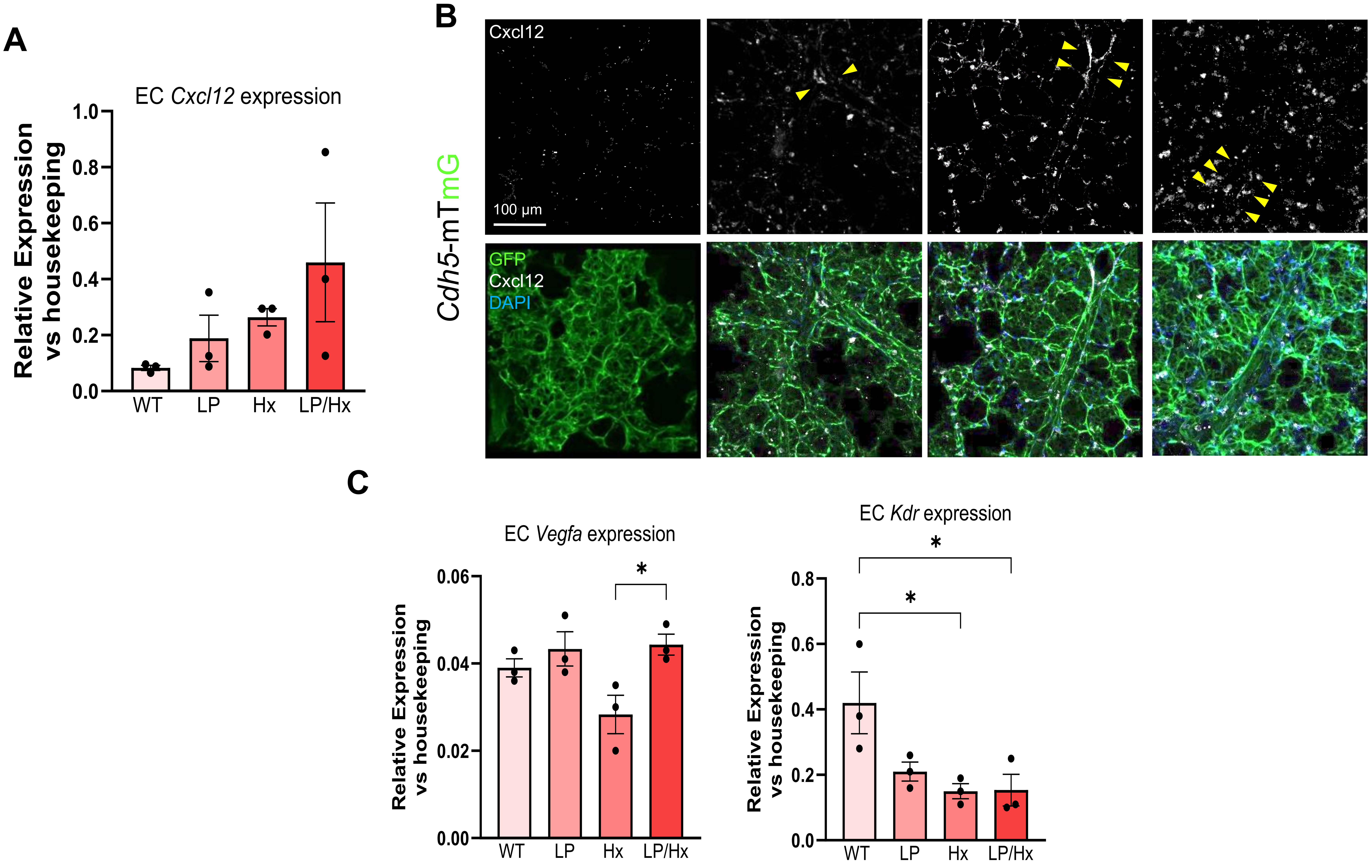

Supplemental Figure 2: Expression of Cxcl12 and angiogenic cell markers in ECs from experimental mice.
A, real-time qPCR (RT-qPCR) of isolated ECs (Cd31+) from control, LP, Hx, and LP/Hx mice for *Cxcl12*. The graph shows the relative expression of *Cxcl12* over the housekeeping gene *B2m.* N=3 for each experimental group.
B, precision cut lung slices (PCLSs) from *Cdh5-mTmG* mice showing increased accumulation of Cxcl12 (white) in *Cdh5* positive cells (ECs) on distal arterioles (yellow arrows) in control, LP, Hx, and LP/Hx mice. Scale bar: 100 µm.
C, rt-qPCR of isolated ECs (Cd31+) from control, LP, Hx, and LP/Hx mice for angiogenic (*Vegfa and Kdr)* cell markers. The graph shows the relative expression of each gene over the housekeeping gene *B2m.* N=3 for each experimental group. Each dot represents a unique sample.
Statistical analysis was performed with one-way ANOVA. Error bars demonstrate mean ± standard error. **p <0.05.*

Supplemental Figure 3

Patient Demographics

Group	Sample #	Age	Gender	Race	Diagnoses	WHO-FC	6MWT (m)	6MWT O2 nadir	MPAP (mm Hg)	PVR (WU)
PAH-CHD (VSD)	1	28	Male	Asian	APAH-VSD	III	484.6	76%	63	NA
	2	68	Male	Asian	APAH-VSD	NA	342.9	89%	84	NA
	3	40	Female	Caucasian	APAH-VSD	IV	280.4	88%	79	23.14
Control	1	57	Female	Unknown	Unknown	NA	NA	NA	NA	NA
	2	81	Female	Unknown	Unknown	NA	NA	NA	NA	NA
	3	68	Male	Caucasian	Cardiac arrest	NA	NA	NA	NA	NA

APAH: associated pulmonary arterial hypertension, PVR: pulmonary pressure vascular resistance units, MPAP: mean arterial pulmonary artery pressure, O2: oxygen, VSD: ventricle septal defect, WHO-FC: World Health Organization functional class, 6MWT: six-minute walk test.

Supplemental Figure 3: Demographic and clinical data from human samples.
Demographic information and clinically relevant data from human PAH-CHD samples (VSD) and non-diseased controls used for SMA and CXCL12 quantifications.

Supplemental Figure 4

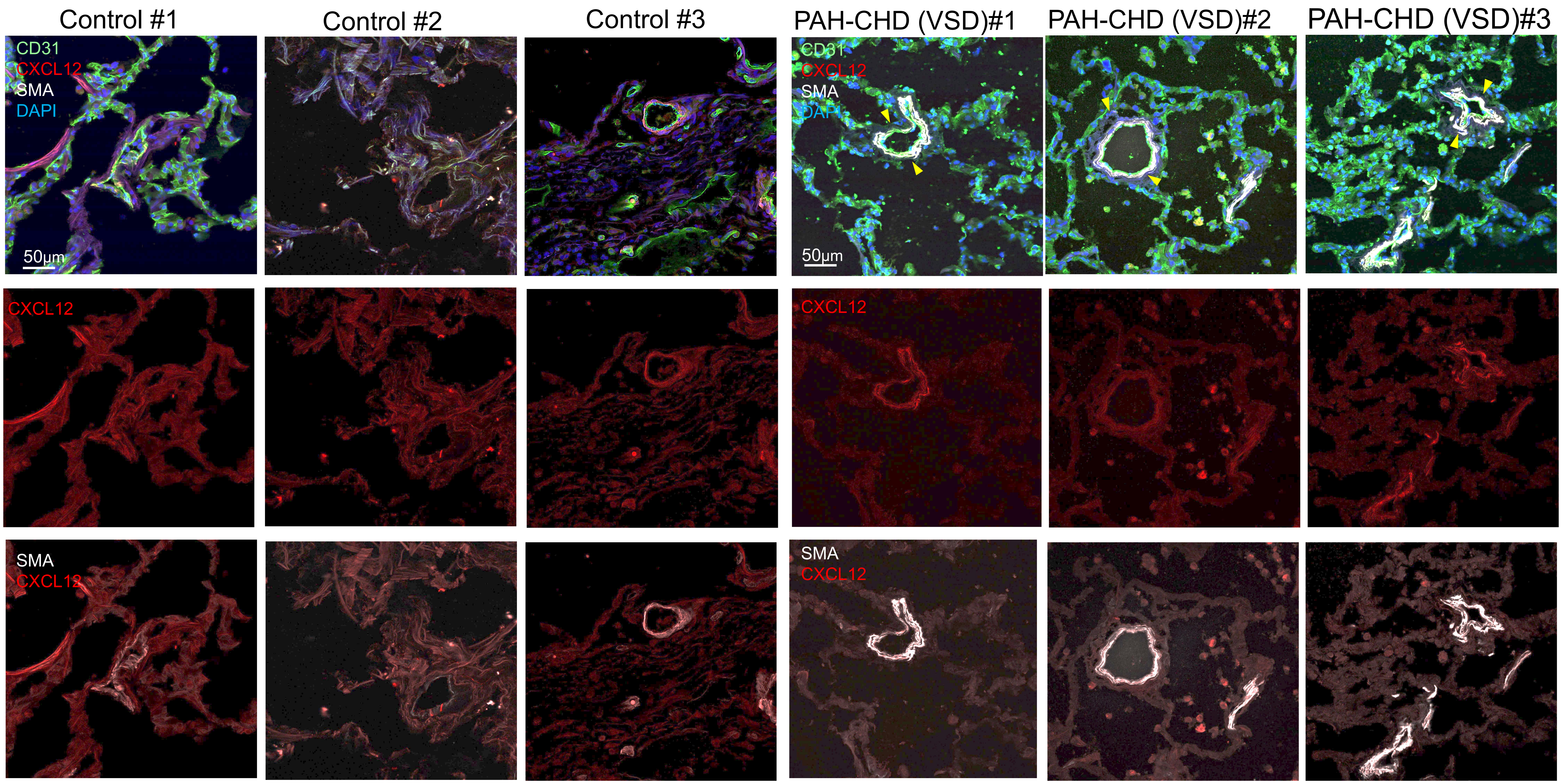

Supplemental Figure 4: CXCL12 staining in PAH-CHD and non-diseased patients.
Staining of lung tissue from three non-diseased patients and three patients with PAH-CHD (VSD) for CXCL12 (red), SMA (white), CD31 (green), and DAPI (blue). Yellow arrows highlight CXCL12 accumulation in remodeled vessels. Scale bar: 100 μm.
