# Supplementary material for: Smooth muscle Cxcl12 contributions to vascular remodeling in flow and hypoxia-induced pulmonary hypertension
Source: J Biol Chem. 2025 May 8;301(6):110207. doi: 10.1016/j.jbc.2025.110207 (PMC12178926; doi:10.1016/j.jbc.2025.110207)
Supplement: Supplemental Videos [file mmc2.pptx]

## Slide 1
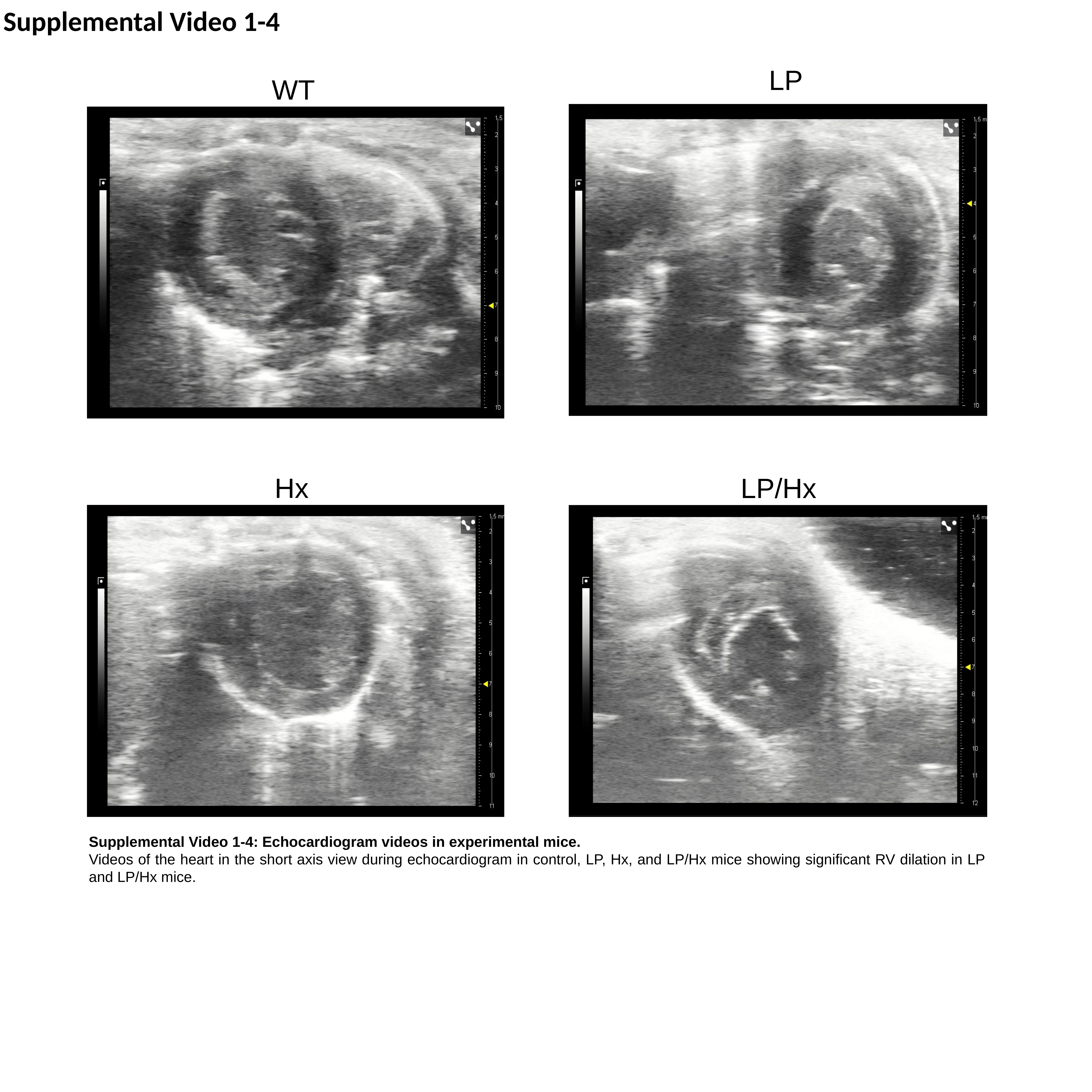

Supplemental Video 1-4
LP
WT
Hx
LP/Hx
Supplemental Video 1-4: Echocardiogram videos in experimental mice.
Videos of the heart in the short axis view during echocardiogram in control, LP, Hx, and LP/Hx mice showing significant RV dilation in LP and LP/Hx mice.
