# Supplementary material for: Smooth muscle Cxcl12 contributions to vascular remodeling in flow and hypoxia-induced pulmonary hypertension
Source: J Biol Chem. 2025 May 8;301(6):110207. doi: 10.1016/j.jbc.2025.110207 (PMC12178926; doi:10.1016/j.jbc.2025.110207)
Supplement: Supplemental Figure 1and video legend [file mmc3.docx]

**Supplemental Figure 1: EC and pericyte fate mapping experiments with *Cdh5-CreERT2::R26-mTmG and Cspg4-CreER™::R26-tdTomato.***

(A) Fate mapping experiments in mice with respective control antibodies (*Cdh5-mTmG:* Cd31 (left), *Cspg4-tdT:* Pdgfrb (middle), and *Acta2-mTmG:* Sma (right)) with endogenous staining (GFP: green, tdT: red). Scale bar: 100µm.

(B) Fate mapping experiments with *Cdh5-mTmG* mice and *Cspg4-tdT* mice in control, LP, Hx, and LP/Hx mice. IF staining was performed for SMA (red) and DAPI (blue). Scale bar: 100µm.

**Supplemental Figure 2: Expression of Cxcl12 and angiogenic cell markers in ECs from experimental mice.**

(A) Real-time PCR of isolated ECs (CD31+) from control, LP, Hx, and LP/Hx mice for *Cxcl12*. The graph shows the relative expression of *Cxcl12* over the housekeeping gene *B2m.* N=3 for each experimental group.

(B) PCLSs from *Cdh5-mTmG* mice showing increased accumulation of Cxcl12 (white) in *Cdh5* positive cells (ECs) on distal arterioles (yellow arrows) in control, LP, Hx, and LP/Hx mice. Scale bar: 100µm.

(C) Real-time PCR of isolated ECs (CD31+) from control, LP, Hx, and LP/Hx mice for angiogenic (*Vegfa and Kdr)* cell markers. The graph shows the relative expression of each gene over the housekeeping gene *B2m.* N=3 for each experimental group. Each dot represents a unique sample.

Statistical analysis was performed with one-way ANOVA. Error bars demonstrate mean ± standard error.

**Supplemental Figure 3: Demographic and clinical data from human samples.**

Demographic information and clinically relevant data from human PAH-CHD samples (VSD) and non-diseased controls used for SMA and CXCL12 quantifications.

**Supplemental Figure 4: CXCL12 staining in PAH-CHD and nondiseased patients.**

Staining of lung tissue from three non-diseased patients and three patients with PAH-CHD (VSD) for CXCL12 (red), SMA (white), CD31 (green), and DAPI (blue). Yellow arrows highlight CXCL12 accumulation in remodeled vessels. Scale bar: 100µm.

**Supplemental Video 1-4: Echocardiogram videos in experimental mice.**

Videos of the heart in the short axis view during echocardiogram in control, LP, Hx, and LP/Hx mice showing significant RV dilation in LP and LP/Hx mice.
